# The pyrazinamide susceptibility breakpoint above which combination therapy fails

**DOI:** 10.1093/jac/dku136

**Published:** 2014-05-12

**Authors:** Tawanda Gumbo, Emmanuel Chigutsa, Jotam Pasipanodya, Marianne Visser, Paul D. van Helden, Frederick A Sirgel, Helen McIlleron

**Affiliations:** 1 Office of Global Health, University of Texas Southwestern Medical Center, Dallas, TX, USA; 2 Department of Medicine, University of Texas Southwestern Medical Center, Dallas, TX, USA; 3 Division of Clinical Pharmacology, Department of Medicine, University of Cape Town, Observatory, Cape Town, South Africa; 4 School of Public Health, University of the Western Cape, Cape Town, South Africa; 5 Division of Molecular Biology and Human Genetics, Stellenbosch University, Tygerberg, South Africa

**Keywords:** anti-tuberculosis drugs, drug susceptibility, MICs, pharmacokinetics, sputum culture

## Abstract

**Objectives:**

To identify the pyrazinamide MIC above which standard combination therapy fails.

**Methods:**

MICs of pyrazinamide were determined for *Mycobacterium tuberculosis* isolates, cultured from 58 patients in a previous randomized clinical trial in Cape Town, South Africa. The MICs were determined using BACTEC MGIT 960 for isolates that were collected before standard treatment with isoniazid, rifampicin, pyrazinamide and ethambutol commenced. Weekly sputum collections were subsequently made for 8 weeks in order to culture *M. tuberculosis* in Middlebrook broth medium. Classification and regression tree (CART) analysis was utilized to identify the pyrazinamide MIC predictive of sputum culture results at the end of pyrazinamide therapy. The machine learning-derived susceptibility breakpoints were then confirmed using standard association statistics that took into account confounders of 2 month sputum conversion.

**Results:**

The pyrazinamide MIC range was 12.5 to >100 mg/L for the isolates prior to therapy. The epidemiological 95% cut-off value was >100 mg/L. The 2 month sputum conversion rate in liquid cultures was 26% by stringent criteria and 48% by less stringent criteria. CART analysis identified an MIC breakpoint of 50 mg/L, above which patients had poor sputum conversion rates. The relative risk of poor sputum conversion was 1.5 (95% CI: 1.2–1.8) for an MIC >50 mg/L compared with an MIC ≤50 mg/L.

**Conclusions:**

We propose a pyrazinamide susceptibility breakpoint of 50 mg/L for clinical decision making and for development of rapid susceptibility assays. This breakpoint is identical to that identified using computer-aided simulations of hollow fibre system output.

## Introduction

Pyrazinamide plays an important role in the sterilizing effect of the treatment of drug-susceptible tuberculosis. This is apparent in its ability to shorten the duration of chemotherapy.^1,2^ The pre-clinical hollow fibre model of tuberculosis has shown that the relationship between pyrazinamide concentration and the sterilizing effect is driven most closely by the 24 h AUC/MIC ratio.^3^ Optimal efficacy was encountered at an AUC/MIC ratio ≥209 at the site of infection in this model. In a recent clinical study of 142 tuberculosis patients, pyrazinamide AUC was found to be the clinical variable most predictive of long-term clinical outcomes.^2^ Patients who achieved a plasma AUC ≥363 mg · h/L had the best outcomes relating to relapse, therapy failure and death. Although the MIC is an integral component of the AUC/MIC index, the isolates in the clinical study were not subjected to pyrazinamide MIC testing. In a separate follow-up clinical study that included pyrazinamide MIC identification, one of the important predictors of the rate of the sterilizing effect in patients was a plasma AUC/MIC ratio of 11.3.^4^ These data translate to a ratio of 201 at the site of lung infection, given the pyrazinamide epithelial lining fluid/plasma ratio of 17.8.^5^ A strong relationship between this AUC/MIC ratio and efficacy was observed and given that treatment failure was also high in patients with AUC values <363 mg · h/L in the prior clinical study, this means that standard short-course chemotherapy is likely to fail if the pyrazinamide MIC exceeds a specific value. That specific pyrazinamide MIC above which therapy fails should be used to define the clinical breakpoint that demarcates patients at higher versus lower risk of therapy failure.

There has been considerable debate regarding the critical concentration or MIC that defines pyrazinamide resistance. Values such as 900, 300, 100 and 64 mg/L were previously proposed in the literature.^6–8^ This is a pressing question for diagnostic laboratories, clinicians and drug susceptibility assay developers. Several international workshops have been recently devoted to this question, such as the ‘Demystifying Pyrazinamide—Challenges and Opportunities’ Workshop 2012 (http://www.newtbdrugs.org/meetings/pza-workshop-2012.php). The standard approach for identifying antibiotic susceptibility breakpoints has been the epidemiological cut-off method. This method is based on the MIC distribution of a drug, which identifies the upper 95% cut-off point on the Gaussian curve.^7–9^ In the past, we used computer-aided clinical trial simulations that relied on the hollow fibre tuberculosis model pharmacokinetic/pharmacodynamic output to identify the breakpoint for pyrazinamide relative to patient outcome.^10^ This was based on the concept that if patients who are receiving the maximum tolerated dose cannot achieve AUC/MICs that are effective at killing *Mycobacterium tuberculosis* in the lungs of patients, then the MICs at which this was observed define clinically meaningful drug resistance. The simulation of 10 000 Western Cape tuberculosis patients suggested a pyrazinamide breakpoint of 25–50 mg/L in Middlebrook broth medium at pH 6.0 for patients that received between 2 and 5 g daily. However, the final arbiter of a resistance breakpoint that has clinical meaning should be clinical studies. Here, we applied an agnostic method to identify the MIC above which standard therapy fails in a clinical study of patients with drug-susceptible tuberculosis in Cape Town.

## Methods

### Study

The primary study was a randomized, double-blinded study that examined the effect of vitamin A and zinc supplementation on the efficacy of the standard combination regimen in the treatment of drug-susceptible tuberculosis.^11,12^ The primary study was approved by the Ethics and Research Committee of the University of Cape Town and registered at http://controlled-trials.com/ISRCTN80852505/80852505. All patients gave their written informed consent. The study was performed at Delft Community Health Center in Cape Town, Western Cape Province, South Africa, between May 2005 and August 2008. Participants were aged between 18 and 60 years and had a positive sputum smear. Exclusion criteria included a history of prior tuberculosis, extrapulmonary tuberculosis, renal or heart failure, elevated alanine aminotransaminase concentrations, corticosteroid use and evidence of pregnancy. Micronutrient supplementation had no effect on sputum culture conversion rates.^11^ Patients also had drug concentrations measured after 1 month of therapy and the results were published.^13,14^ However, the peak concentrations and AUCs achieved were also used in the current study to compare drug exposures in patients infected by *M. tuberculosis* with pyrazinamide MICs above or below the identified susceptibility breakpoints.

### Sputum processing and pyrazinamide susceptibility testing

Smear examination by fluorescence microscopy and culture by standard BACTEC MGIT 960 methods were done on initial and weekly sputum collections for a period of 8 weeks. The identity of *M. tuberculosis* was confirmed by PCR and the isolates were kept at −80°C after the study was completed. Spoligotyping was also performed on the isolates during the study; the isolates were not from clusters and 44% belonged to the W-Beijing genotype.^12^ For the current substudy, viable isolates were used from the archived (−80°C) collection. Susceptibility testing was done on these isolates against pyrazinamide, rifampicin, ethambutol and isoniazid at Stellenbosch University, Western Cape.

MICs of pyrazinamide were then determined for each of the selected clinical *M. tuberculosis* isolates with the automated BACTEC MGIT 960 system equipped with EpiCenter software and the TBeXist application (Becton Dickinson), as previously described.^15^ Pyrazinamide is only active against *M. tuberculosis* in an *in vitro* culture at a lower pH. BACTEC MGIT 960 PZA Medium tubes with an adjusted pH of 5.9 were therefore used in this study. BACTEC MGIT 960 lyophilized pyrazinamide was reconstituted in 2.5 mL of sterile distilled water, as described by the manufacturer to obtain a stock solution of 8000 mg/L. Serial 2-fold dilutions were then prepared and 100 μL of each dilution was transferred to the corresponding MGIT tube to give final drug concentrations of 12.5, 25, 50 and 100 mg/L. The drug-free control vial was inoculated with a 1 : 10 dilution of the inoculum to represent 10% of the bacterial population. *M. tuberculosis* H37Rv (ATCC 27294) was used as the reference standard.

### Outcomes

The primary outcome was the 2 month sputum culture conversion. Patients were considered to have failed to convert if week 7 and 8 sputum samples were positive according to liquid cultures, as described in previous clinical trials.^16^ The secondary outcome used a less stringent definition and was based on a single sputum culture at the 8 week timepoint. The 2 month sputum conversion was chosen as an outcome for two reasons: (i) this parameter is an early indicator of clinical outcome and sterilizing activity;^17,18^ and (ii) it measures sputum culture conversion during the initial 2 month treatment period while the regimen contains pyrazinamide. The MICs of pyrazinamide were thus determined and examined as a predictive marker of clinical outcome.

### Classification and regression tree (CART) analyses and cross-validation

We utilized CART analysis to identify the MIC cut-off predictive of 2 month sputum culture conversion. CART analysis is a non-parametric method that uses binary recursive partitioning to assign patients to homogenous groups and then present results in the form of intuitive and easy-to-interpret decision trees.^19^ CART analysis searches through potential predictors and all possible cut-off values of the variables to identify the best predictor for classifying between patients with and without the outcome. This results in an upside-down ‘tree’ whose root node is the primary predictor. We utilized the Gini criterion function for splitting nodes and attaining the minimum cost tree. The resulting trees were pruned to improve the predictive value of the models by using the receiver operator curve (ROC) score of both the training and test samples. The optimal trees were then chosen based on relative misclassification costs, complexity and parsimony. We performed 5-fold validation of the results. In the cross-validation, the dataset was randomly split into learning and test databases and CART analysis performed; this was repeated five times. CART analyses were performed using Salford Predictive Miner System software (San Diego, CA, USA). We report the ROC score of the test sample.

### Statistical comparisons

Clinical factors, such as low antibiotic AUC and peak concentrations, bacillary burden at diagnosis (we utilized time to positivity of sputum cultures at diagnosis), presence of cavities on chest X-rays and age are also risk factors of poor 2 month sputum conversion.^2,12,20^ We wanted to take these into account, in order to further delineate the role of the CART analysis-derived susceptibility breakpoint. First, we examined the 2 month sputum conversion outcomes between patients whose isolates had MICs above the CART analysis-derived breakpoint versus those with MICs at or below the breakpoint. These variables were thus compared among the two groups of patients comprising those with isolates above the CART analysis-derived MIC breakpoint versus the rest. Continuous variables were tested for differences between the groups using Student's *t*-test or the Mann–Whitney *U*-test when applicable. Categorical variables were tested for differences using Pearson's *χ*^2^ test. A two-tailed *P* value of ≤0.05 was considered statistically significant. All statistical tests were run in GraphPad version 5 (GraphPad Software, CA, USA).

## Results

**Figure 1.**
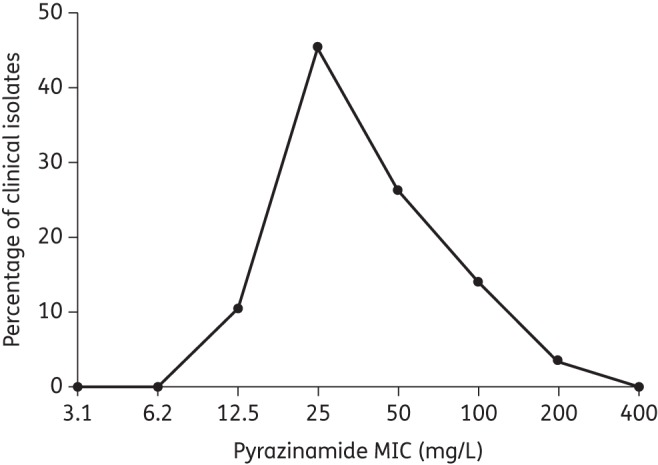
Pyrazinamide MIC distribution in 58 tuberculosis patients. Distribution of MICs, based on inputting all values >100 mg/L as 200 mg/L. As a result, the range goes up to 400 mg/L, which would have a value of 0% of clinical isolates.

Altogether, 58 patients [37 (64%) men and 21 (36%) women] had pyrazinamide MICs identified for the initial isolate at diagnosis, before therapy commenced. The median (range) age and body mass index were 26.5 (17–55) years and 19.12 (14.37–27.89) kg/m^2^, respectively. Eight (14%) patients were HIV co-infected and 91% of the patients had cavities on chest X-ray. All patients in the study had pyrazinamide susceptibility tests performed. The pyrazinamide MIC for the standard laboratory strain, *M. tuberculosis* H37Rv, was 50 mg/L on each of two different occasions. The MIC distribution among the clinical isolates at the time of diagnosis is shown in Figure 1. The median MIC was 25 mg/L, with a range of 12.5 to >100 mg/L. The mean (and standard deviation) MIC was 46.55 ± 39.65 mg/L when the two isolates with an MIC >100 mg/L were imputed as 200 mg/L. The distribution has a right skew. The epidemiological 95% cut-off value was >100 mg/L. The isolates were susceptible to isoniazid, rifampicin and ethambutol based on both drug susceptibility testing at the time of recruitment and the MICs of each of these drugs.

**Figure 2.**
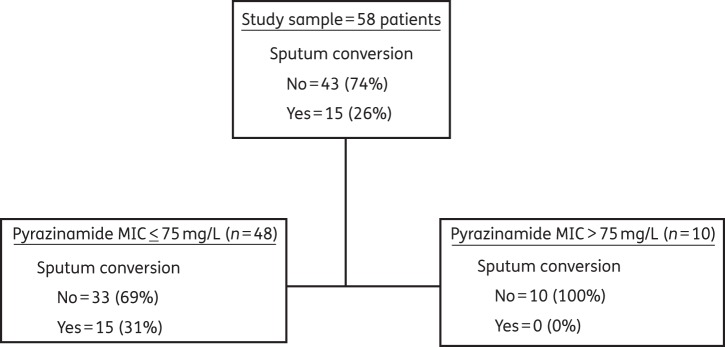
CART analysis for primary outcome. The primary node, based on the stringent definition of 2 month sputum conversion, shows that the MIC above which therapy fails is 75 mg/L.

The 2 month sputum conversion rates using the more stringent criteria of negative sputum cultures in liquid media at both 7 and 8 weeks was only 26%. The CART analysis results of the relationship between the MIC and sputum conversion are shown in Figure 2, which is the optimal tree for the primary outcome. Figure 2 shows that the MIC cut-off value was ≤75 mg/L. The ROC score was 0.63. The results of 5-fold cross-validation with the test datasets gave an identical optimum tree and MIC cut-off values of ≤75 mg/L. Rounding to the nearest 2-fold dilution MIC value <75 mg/L gives a susceptibility breakpoint of 50 mg/L. If the results are examined using standard association statistics, the relative risk of having a positive sputum in patients with an isolate with an MIC >50 mg/L compared with those with isolates that had an MIC ≤50 mg/L was 1.5 (95% CI: 1.2–1.8; *P* = 0.04). Since several factors affect the 2 month sputum conversion rates and are therefore confounders, we compared their distribution between those with an MIC >50 mg/L and those without (results shown in Table 1). The two groups of patients had very similar risk factors for failure, such as age, chest X-ray cavities, bacterial burden (as measured by time to positivity), HIV status and drug AUC and peak concentrations. Therefore, differences in 2 month sputum conversion can be attributed to the CART analysis-derived susceptibility breakpoints, since all other confounders where similar between the groups except the MICs.

**Table 1.** Comparison of risk factors of 2 month sputum culture conversion between patients with isolates that had MIC above and below the CART analysis-derived susceptibility breakpoint (75 mg/L)

	MIC > breakpoint (*n* = 10)	MIC ≤ breakpoint (*n* = 48)	*P* value
Variable			
age (years)	28.60 ± 10.22	31.10 ± 11.09	0.54
gender (% males)	70.00	62.50	0.73
HIV infection (%)	10.00	14.58	1.00
weight (kg)	51.29 ± 9.12	53.73 ± 7.98	0.22
chest X-ray cavities present (%)	100	89	1.00
time to positivity of culture at diagnosis (days)	10.78 ± 9.20	9.28 ± 4.40	0.56
Drug concentrations and AUCs			
pyrazinamide AUC (mg · h/L)	429.90 ± 95.96	438.80 ± 140.10	0.91
pyrazinamide peak (mg/L)	34.85 ± 6.15	33.93 ± 8.78	0.51
rifampicin AUC (mg · h/L)	48.04 ± 20.87	51.62 ± 23.15	0.60
rifampicin peak (mg/L)	7.11 ± 1.88	7.50 ± 1.85	0.44
isoniazid AUC (mg · h/L)	12.79 ± 4.03	20.41 ± 13.81	0.13
isoniazid peak (mg/L)	2.71 ± 1.25	2.86 ± 1.86	0.94
ethambutol AUC (mg · h/L)	30.03 ± 11.33	34.38 ± 14.96	0.41
ethambutol peak (mg/L)	2.73 ± 0.84	3.060 ± 0.90	0.29

**Figure 3.**
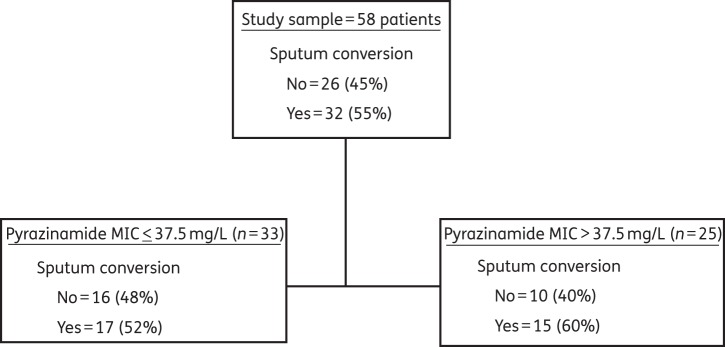
CART analysis for secondary outcome. The MIC above which therapy fails, based on a less stringent definition of 2 month sputum conversion was >37.5 mg/L.

If one considers the secondary outcome, which was sputum culture conversion based on only one negative sputum culture at 8 weeks, then the sputum conversion rate was 48%. The optimal tree chosen using those outcome criteria is shown in Figure 3. In this case, the root node selected an MIC of 37.5 mg/L as the threshold value. The test tree was similar, but was deeper and contained daughter nodes with a further MIC cut-off of ≤18.75 mg/L for those with an MIC ≤37.5 mg/L, and ≤75 mg/L for those with an MIC >37.5 mg/L. The primary node translates to 17/33 (51.52%) patients with isolates at ≤25 mg/L who continued to have positive sputum cultures versus 15/25 (60%) who had an isolate with an MIC >25 mg/L (*P* = 0.432). Thus, this second lower breakpoint was not confirmed by statistics employing measures of association and was not considered further.

## Discussion

The intent of chemotherapy is to cure patients. An important aspect of susceptibility tests in the medical microbiology laboratory is to give guidance as to which patient is likely to respond to a particular drug in the chemotherapy regimen. We utilized CART analyses to identify the pyrazinamide MIC breakpoint predictive of poor outcome at the end of pyrazinamide therapy. The MIC identified was 50 mg/L at pH 5.9. Previously, we employed a hollow fibre model of tuberculosis output, and the pharmacokinetic variability encountered in patients from the Western Cape, in computer-aided clinical trial simulations to identify a susceptibility breakpoint.^3,10,21^ We found that the breakpoint MIC in patients receiving a pyrazinamide dose of 2–4 g a day was 25 mg/L without adjustment for body weight, but 50 mg/L with weight adjustment (i.e. between 25 and 50 mg/L). These values are the exact ones identified by CART analysis, an agnostic method, with the primary and secondary outcomes in the current study. However, the susceptibility breakpoint of 50 mg/L was associated with statistically significant differences regarding outcomes in patients on standard therapy and is therefore recommended for laboratory use. This value is close to the 64 mg/L proposed by Werngren *et al.*^7^ based on MIC distributions for isolates in Europe. Nevertheless, our current data, especially those presented in Figure 1, strongly suggest that these distributions vary from locale to locale.

An examination of the literature could also help to suggest the approximate range for the pyrazinamide MIC susceptibility breakpoint. We have found that a pyrazinamide plasma AUC of 363 mg · h/L is associated with optimal sterilizing effect in patients, while an AUC/MIC ratio of 209 in the lung was associated with optimal sterilizing effect in pre-clinical models.^2,3^ The plasma AUC of 363 mg · h/L translates to 7986 mg · h/L in the lung, at most, given a lung penetration ratio of 17.8–22.^5^ This means the maximal MIC that leads to optimal efficacy in the lung, given that the pyrazinamide AUC/MIC of 209 is associated with optimal microbial kill, translates to ∼40 mg/L. Increases in the MIC above this lead to greater decreases in the AUC/MIC ratio. This is more so if one considers the population pharmacokinetics of pyrazinamide identified in the largest studies published.^21,22^ As shown in Table 2, even with the best-case scenario of a penetration ratio of 22 (which is overgenerous), the AUC/MIC falls dramatically below the optimal ratio between 25 and 50 mg/L in the majority of patients and is below the optimal ratio in the majority as the MIC increases above 50 mg/L. Another view, which takes only plasma concentrations into account, would be to consider that if the plasma AUC that predicts poor outcome is 363 mg · h/L^2^ and the plasma AUC/MIC ratio that predicts failure is 11.3,^4^ then the MIC above which therapy fails would be approximately 32 mg/L. These simple exercises further suggest that the susceptibility breakpoint of 100 mg/L is too high and that it should be ∼50 mg/L. This value is close to the mean MIC; thus, half of the patients in our current study would be considered to have isolates with pyrazinamide resistance. Since higher pyrazinamide doses would lead to higher AUCs, it is conceivable that with larger doses a higher susceptibility breakpoint could be identified in the future.

**Table 2.** Pyrazinamide AUC/MIC in serum/plasma, corresponding estimated AUC/MIC in lung, and AUC/MIC achieved in lung of ≥50% of patients at each MIC

Reference	Median pyrazinamide AUC (mg · h/L)		Pyrazinamide MIC (mg/L)			
	serum/plasma	lung	12.5	25	50	100
Zhu *et al.*^22^	368	8096	647.68	323.84	161.92	80.96
Wilkins *et al.*^21^	451	9922	793.76	396.88	198.44	99.22

There are currently concerted efforts to develop assays that can rapidly identify patients who would fail pyrazinamide therapy. Most effort has focused on finding mutations in the pyrazinaminidase-encoding genes (*pncA*) and developing faster molecular assays.^23^ However, other mechanisms of pyrazinamide resistance beyond *pncA* are known to be important, e.g. efflux pumps. We have proposed that efflux pump induction is an early event in the process that will lead to chromosomal mutations, making the two mechanisms integral to each other.^24^ Zimic *et al*.^25^ have demonstrated that the efflux rate of pyrazinamide's active moiety, pyrazinoic acid, predicts pyrazinamide resistance with up to 93% sensitivity and 100% specificity, which is better than *pncA* mutations. Indeed, pyrazinoic acid efflux accounted for 61% of the variability in pyrazinamide susceptibility.^26^ Therefore, resistance mechanisms other than *pncA* mutations contribute to pyrazinamide resistance. Our new pyrazinamide breakpoint was derived using a laboratory phenotypic test and patient response, which do not depend on mechanisms of effect, i.e. it should identify drug resistance regardless of mechanism. On the other hand, the susceptibility breakpoint we identified will bisect the Gaussian curve of the MIC distribution, violating rules for the epidemiological cut-off method of setting susceptibility breakpoints.^27^ This proscription is based on the understanding that the ‘methodological variation [in susceptibility testing] obscures any biological variation in these distributions. The biological variation among strains lacking resistance mechanisms is too small to be detected with ordinary MIC testing and in all probability too small to be clinically utilizable.’^27^ Perhaps, a better solution will be to design assays that are more sensitive with little interoccasion variability. However, our own findings will need to be validated in larger prospective clinical studies.

Our study has several potential limitations. First, the study was performed in the Western Cape, where, like in most African study sites, response rates are poorer than in Western countries.^28^ This may limit generalizability of the results. Second, our study population was of 58 patients, which could also limit generalizability. However, CART analysis has been able to identify predictive concentration thresholds in even smaller populations on combination therapy with other anti-infective agents in the past.^29^ Third, several other parameters predict clinical outcomes in the treatment of tuberculosis, including drug concentrations, bacterial burden, chest X-ray findings of cavitation and HIV infection.^2,4,12,20,30^ Thus, the pyrazinamide MIC is not the only factor that contributes to outcome. Nevertheless, we demonstrated that patients with isolates with MICs >50 mg/L had the same peak and AUC concentrations of all drugs, similar bacterial burdens and the same proportion of patients with chest X-ray cavities and HIV infection, compared with those with isolates with MICs ≤50 mg/L. Thus, failure of therapy could still be attributed to the pyrazinamide MIC being >50 mg/L.

In summary, we utilized CART analysis to identify a pyrazinamide MIC that could separate patients at higher risk of therapy failure. The pyrazinamide MIC of 50 mg/L was identified. This is the critical concentration that should be used to identify resistant isolates for the purposes of clinical decision making.

## Funding

This work was funded by grants from: the Clinical Infectious Diseases Research Initiative (CIDRI) Wellcome Trust Fund 412164; the National Research Foundation (NRF) South Africa (2067444 and RCN 180353/S50); the Norwegian Programme for Development, Research and Higher Education (NUFUPRO-2007/10183); the Research Council of Norway (RCN) (183694/S50); the South African Medical Research Council; and the National Institute of Allergy and Infectious Diseases of the National Institutes of Health (R01AI079497). The funding agencies were not involved in the: design and conduct of the study; collection, management, analysis and interpretation of data; and preparation, review or approval of the manuscript.

## Transparency declarations

T. G. has received research grants from Merck for work on antifungal agents. T. G. has worked as a consultant for Astellas Pharma US Inc. on antifungal work. T. G. founded Jacaranda Biomed Inc. All other authors: none to declare.
